# Effect of support based on family centered empowerment model on care burden in family caregivers of patients with multiple sclerosis

**DOI:** 10.3389/fpubh.2023.1115311

**Published:** 2023-07-13

**Authors:** Farshid Mohammad Mousaei, Seyedmohammad Mirhosseini, Mohammad Hossein Mafi, Nevin Günaydın, Hamid Reza Zendehtalab

**Affiliations:** ^1^School of Nursing, Abadan University of Medical Sciences, Abadan, Iran; ^2^Department of Nursing, School of Nursing and Midwifery, Shahroud University of Medical Sciences, Shahroud, Iran; ^3^School of Nursing and Midwifery, Research Institute for Prevention of Non – Communicable Diseases, Qazvin University of Medical Sciences, Qazvin, Iran; ^4^Department of Psychiatric Nursing, Health Sciences Faculty, Ordu University, Ordu, Turkey; ^5^Department of Community Health of Nursing, School of Nursing and Midwifery, Mashhad University of Medical Sciences, Mashhad, Iran

**Keywords:** caregiver burden, family caregivers, family-centered empowerment model, multiple sclerosis, nursing care

## Abstract

**Introduction:**

Family caregivers of patients with multiple sclerosis (MS) are at risk of care burden that may lead to a detrimental effect on their quality of life (QoL), physical and mental well-being. This study aimed to determine the effect of the family-centered empowerment model (FCEM) on the care burden of caregivers of patients with MS.

**Methods:**

This quasi-experimental study was conducted using convenience sampling on 60 caregivers of patients referring to the Multiple Sclerosis Clinic in Ghaem Hospital, Mashhad, Iran. The participants were assigned to FCEM and control groups based on the days they were referred to the MS clinic. Data collection tools included the Zarit Caregiver Burden Inventory (CBI), completed in the intervention and control groups before and 1 month after the intervention. The support based on FCEM was provided during eight 45-60-min sessions, and the control group received the medical center’s routine training. Data were analyzed by Chi-square, independent *t*-test, analysis of covariance, and repeated measure tests.

**Results:**

The results of the present study showed that all demographic characteristics were homogeneous at the baseline. Before the intervention, no significant difference was observed between the two groups regarding mean scores of care burden. Based on the repeated measure test, there was no significant treatment and time interaction in changes in care burden.

**Conclusion:**

The FCEM has no significant effect in alleviating the care burden. It is recommended to observe the necessary considerations regarding the context of this type of intervention and to carry out further investigations in different intervals.

## 1. Introduction

Multiple sclerosis (MS) is recognized as a neurodegenerative and demyelinating disease related to the immune system (1). The number of people with MS has increased to 2.8 million, i.e., by 30% compared to 2013. The prevalence of this disease is estimated at 35.9 per 100,000 people (2) in the world and 29.3 per 100,000 in Iran (1). The disease typically results in severe physical or cognitive disability in patients besides neurological problems (3). In addition, a large number of patients continue to live independently, but some need constant care and support. So, family members often have to provide this support and play the role of “informal caregivers” (4).

A literature review reveals that family caregivers of patients with MS are considerably exposed to the risk of a “care burden” (5). Imposed care burden may devastate the caregiver’s quality of life (QoL), physical and mental well-being, and financial and employment status (6). Caring for these patients is a severe challenge for informal caregivers, so most feel helpless and frustrated. In the meantime, adherence to supporting caregivers eliminates their feeling of failure (7).

Moreover, providing some support to the patient necessitates the development of the caregiver’s new knowledge and skills, conducted by giving them training, support, and empowerment (8). Using the family-centered empowerment model (FCEM) is a crucial nursing measure to affect family caregivers (9).

As a model based on the family-centered care philosophy, the FCEM simultaneously promotes the patient and family’s knowledge, skills, values, and beliefs (10, 11). Alhani et al. introduced the Family Centered Empowerment Model (FCEM). This model has been presented as one of the models appropriate to the culture and structure of health in the Iranian community to empower patients or their family caregivers, with the aim of providing support based on the description of essential factors to improve the outcomes of chronic patient care (12–14). The main action in this model is to involve the clients and their families in making decisions to improve their health. In this regard, the results of other studies reveal the positive effect of applying this model in improving the QoL (15), self-efficacy (16), self-concept, and perceived satisfaction (7) of caregivers and patients with MS. Generally, the nursing interventions in family-centered care aim to enhance the family members’ abilities in specific areas to overcome barriers in the health and wellness fields (17). To better support caregivers of these patients, research should be conducted to improve the QoL besides reducing the care burden. Thus, based on the scientific evidence that revealed the effectiveness of the FCEM on other health-related variables, this model seems to positively impact the care burden of caregivers of patients with MS. The present study aimed to determine the effect of the FCEM on the care burden of caregivers of patients with MS.

## 2. Materials and methods

### 2.1. Participants

The current study was conducted in a quasi-experimental design among caregivers of patients with multiple sclerosis in the MS Clinic of Ghaem Hospital, Mashhad, Iran. Participants were selected using the convenience sampling technique. Inclusion criteria were the minimum literacy level for completing the questionnaires in caregivers, not participating in similar educational support programs, at least 6 months of care for a patient with MS, no psychiatric disorders, and neuroleptic medication. Exclusion criteria were considered, such as acute physical or mental traumas during the study, death, patient transfer to other medical centers, and absence of more than one session in the training sessions. The assignment procedure was based on non-randomized allocation and it was conducted based on referral days to the MS clinic. The current research was carried out between February and June 2022.

### 2.2. Intervention

After obtaining the required permissions, intervention sessions based on FCEM were held at the patient education department of the MS Clinic of Ghaem Hospital, Mashhad, Iran. The intervention was performed as support based on the FCEM. The main goal of the model is to empower the patient or his family to improve the level of health. The model has four steps: (a) determination of perceived threat (group discussion method); (b) self-efficacy (problem-solving method); (c) Improvement of self-esteem (educational participation method), and (d) process and outcome assessments (12). The FCEM was performed based on the steps described in Table 1. The implementation of the intervention in this study was performed by the first author, who is an expert community health nurse. The intervention was performed for caregivers during a face to face sessions in groups of six caregivers (in lectures, group discussions, questions and answers, practical demonstrations, and brainstorming), in eight sessions of 45–60 min during eight weeks (one session during the week). The present intervention was based on one of the important models introduced in Iranian culture, specially introduced to empower patients/their families. The context of the intervention and the provided training has been personalized-tailored and implemented with the background of MS disease, its consequences, and the needs of caregivers of MS patients. In this study, the psychological strategies that guided the intervention focused on self-efficacy and self-esteem. The following strategies were used to improve these two factors: (1) Patients and caregivers were justified that they could change their lifestyle. (2) Family caregivers were invited to design a care plan based on empowering patients. (3) Caregivers were asked to share their successful experiences caring for patients in group discussion sessions. Then the positive aspects of these experiences were emphasized, and other caregivers were asked to use these experiences in their daily practices while caring for the patient. (4) During a friendly interaction, caregivers could ask and answer questions and express their care challenges and problems. (5) The appropriate images, slides, and educational videos were provided in addition to the practical training to show the care technique to create deeper learning. (6) During the training presentation, the caregivers were asked to carry out the training in practice in the presence of the researcher and then identify their limitations and strengths points. (7) Caregivers were encouraged to perform care correctly, and if there were deficiencies in their actions, they were asked to practice more. (8) A follow-up program was implemented to have a continuous relationship with the caregivers. In this way, the phone number of one of the researchers was provided to the participants so that they could call in case of a specific problem or if they have any questions or guidance (12). Given the COVID-19 pandemic, intervention sessions were held according to the observance of health protocols and social distancing, and provided personal protective equipment for study participants. All of the information on intervention was provided based on the TIDieR checklist used for reporting and reproducibility (18).

**Table 1:** Description of sessions.

	Topics	Content of sessions
1	Perceived threat	Images of patients who had complications in the neurology ward due to lack of self-care were shown to caregivers to create a perceived threat. In addition, patients were visited twice, and their caregivers were interviewed to familiarize the participants with the problems and burdens on caregivers due to disease mismanagement and non-compliance with self-care behaviors.
2	Self efficacy	Patients discussed, discussed, and exchanged experiences with each other under the direct and direct supervision of the researcher, citing concrete examples of their condition.
		To create self-efficacy, some of the patients’ self-care behaviors include getting out of bed, taking a bath, and aerobic exercise. The researcher performed the practical demonstration of Kegel exercises step by step (and then practiced by patients).
3	Self esteem	The patient and the caregiver have taught 1–2 patients or other caregivers what they have learned in the presence of the researcher.
		Encourage the patient and caregiver if they provide proper education to patients or other caregivers
4	Evaluation	At the beginning of each session, the stages of the family-centered empowerment model (perceived threat, self-efficacy, self-esteem) were evaluated. During the intervention process, learning the content of previous sessions was assessed by asking questions about the illness and care learned in the training classes. The training provided was followed by two phone calls during the week.

Furthermore, the routine training in the clinic was provided in educational pamphlets for the control group during the study process. After the study, the mentioned content was delivered in a group form in the control group.

### 2.3. Outcomes

Data collection tools included a demographic information questionnaire and Zarit caregiver burden inventory completed before and 1 month after the intervention. The form included demographic information, i.e., age, length of care, sex, marital status, education, relationship with the patient, and occupational status.

The care burden was considered the primary outcome of the present research. The caregiver burden inventory was designed by Zarit et al. (19) to specify the level of care burden, including 22 questions about the burden imposed by a caring a patient on the caregiver and the responses are based on Likert scale (never = 0, rarely = 1, sometimes = 2, often = 3, and always = 4). The sum of the scores obtained by each caregiver specifies his/her psychological burden. A score of less than 30 is considered as a low, 31 to 60 as moderate, and 61 to 88 as severe care burden. The minimum and maximum scores obtained by each person in this tool are 0 and 88, respectively. A higher score indicates greater levels of care burden (19). Smith and Schwirian determined the reliability coefficient of this questionnaire by the test–retest method (20). Navidian et al. (21) reported the reliability coefficient of this tool to be 0.94 using the retest method (21). In the current study, the reliability was calculated using the internal consistency method and by Cronbach’s alpha coefficient equal to 0.85.

### 2.4. Sample size

Based on the previous study (22), according to mean scores of care burden in the control (*M* = 15.78, SD = 9.71) and intervention (*M* = 27.92, SD = 9.85) groups, given the 95% confidence level and 90% statistical test power considering the probability of sample loss, the sample size was determined to be 30 (60 subjects in both groups). In the present study, data collectors and statistical consultants were blind, but due to the nature of the intervention, it was impossible to blind the participants. The unit of assignment was at an individual level. A total of 60 caregivers attended the clinic regularly during the study period, those who came on even days were assigned to the FCEM group, and those who came on odd days were assigned to the control group.

### 2.5. Data analysis

Caregivers, as the smallest unit, were analyzed. Descriptive data were provided by frequency, percentage (to present the descriptive data for gender, marital status, educational level, occupational status, and relationship with a patient), mean, and standard deviation (SD) (to present the descriptive data for age and care burden scores). In order to present the difference between the two groups, Chi-squared and the Exact Fisher tests were utilized (In variables where the number of observations was less than 20 or the number of observations was between 20 and 40 and the smallest expected frequency was less than five, Fisher’s test was used. Also, the Chi-squared test was used for the variables that had a maximum of 20% expected frequency less than five). Also, to show the difference between the two groups in mean scores of care burden at the baseline, the independent sample t-test was used. Finally, to compare the care burden scores after the intervention between the control and FCEM groups, while considering and eliminating the effect of the confounding variables (such as pretest factor), the analysis of covariance (ANCOVA) model was used. To assess the time and treatment interaction for the main variable (care burden), the repeated measure test was utilized. The significance level was considered at 0.05. All statistical analyses were performed using Statistical Package for the Social Sciences (SPSS) software.

### 2.6. Ethical considerations

This study has been approved by the Ethics Council in Biomedical Research of Mashhad University of Medical Sciences (IR.MUMS.NURSE.REC.1399.087). Participation in the research was voluntary, and the participants were informed about the research objectives and the confidentiality of information. The authors adhered to Committee on Publication Ethics (COPE) principles in publishing the results. The analysis and publication of the results were conducted anonymously and with observing the publishing ethics considerations. All participants were informed about the study procedures and provided informed consent to participate.

## 3. Results

The present study results revealed that the mean age of caregivers in the intervention and control groups were (*M* = 39.1, SD = 13.4) and (*M* = 38.3, SD = 12.3), respectively. No significant difference was observed between the two groups regarding demographic variables such as age, sex, marital status, education level, occupational status, and relationship with a patient. In this regard, they were homogeneous (*p* < 0.05) (Table 2).

**Table 2:** Demographic characteristics of caregivers.

Variables		Groups		*p*-value
		Intervention *n* (%)	Control *n* (%)	
Gender	Male	19 (63.3)	20 (66.7)	0.78^*^
	Female	11 (36.7)	10 (33.3)	
Marital status	Single	22 (51.5)	19 (48.5)	0.34^**^
	Married	4 (48.6)	8 (51.4)	
	Divorced	2 (6.7)	3 (10.0)	
	Deceased wife	2 (6.7)	0 (0.0)	
Level of education	Illiterate	0 (0.0)	2 (6.7)	0.41^**^
	Elementary school	2 (6.7)	1 (3.3)	
	Secondary school	4 (13.3)	5 (16.7)	
	Diploma	12 (40.0)	15 (50.0)	
	Academic degree	12 (40.0)	7 (23.3)	
Employment status	Housewife or unemployed	5 (16.7)	6 (20.0)	0.84^**^
	Self-employed	12 (40.0)	15 (50.0)	
	Retired	4 (13.3)	3 (10.0)	
	Employee	9 (30.0)	6 (20.0)	
Relationship with the patient	Mother	6 (20.0)	1 (3.3)	0.32^**^
	Sister	3 (10.0)	3 (10.0)	
	Brother	2 (6.7)	2 (6.7)	
	Father	2 (6.7)	4 (13.3)	
	Son	2 (6.7)	4 (13.3)	
	Spouse	15 (50.0)	16 (53.3)	

*Chi-squared test. **Fisher’s exact test. *n*, frequency; %, percent; SD, standard deviation.

Based on the results of present study, the mean scores of care burden in the FCEM and control groups were (*M* = 24.2, SD = 12.3) and (*M* = 25.6, SD = 10.3), respectively. Before the intervention, no significant difference was observed between the two groups regarding the mean scores of care burden (*p* = 0.62). In addition, the results of the ANCOVA showed that the pre-intervention mean score of care burden and group variables were effective on the post-intervention mean score of care burden. Also, the participants in the control group reported a higher care burden score of 3.645 units than the FCEM group (Table 3).

**Table 3:** Assessment of the efficacy of FCEM on care burden after eliminating the effect of pre-test scores.

Variables		β	SE	*t*	*p*-value
Constant value		5.754	1.470	3.913	<0.001
Care burden (before intervention)		0.629	0.051	12.361	<0.001
Group	Intervention	Ref.			
	Control	3.645	1.140	3.196	0.002

FCEM, Family centered empowerment model; SE, standard error.

Regarding the mean scores of care burden, the results of Muchly’s test showed that the hypothesis of sphericity is not satisfied (*p* < 0.05), therefore, the Greenhouse–Geisser test was considered. This test showed that time was effective in the changes of care burden scores (*p* < 0.036) and the interaction between time and treatment was not significant (*p* < 0.053). On the other hand, there is no statistically significant difference between the two groups in terms of the care burden mean scores (*p* < 0.225) (Table 4).

**Table 4:** Efficacy of FCEM on the care burden using repeated measure to assess the time and group interaction.

Variable		Mean square	*F*	*p*-value
Care burden	Time	85.008	4.602	0.036
	Group	273.008	1.506	0.225
	Time * Group	72.075	3.902	0.053

FCEM, family centered empowerment model. The care burden was measured twice times (pretest, posttest).

## 4. Discussion

It should be noted that besides affecting patients, MS emotionally, socially, economically, and physically affects their caregivers. In this regard, the results of previous studies showed that they imposed significant levels of care burden (23–25). According to the findings of previous studies, caregivers of patients with MS reported high levels of stress, anxiety, and negative emotions, and also few researchers have implemented suitable psychological and training interventions for these caregivers (26, 27), so the previous researchers have advised identification and meeting the needs of MS caregivers as the best way to provide psychosocial support (6). The dominant conceptual model for caregiving assumes the onset and progression of chronic diseases and physical disabilities like MS are stressful for both the patients and the caregivers. This context presents the objective stressors, including physical disabilities, self-concept disorders, problem behaviours, and the type and intensity of care. These objective stressors result in psychological distress and the care burden given the tedious duties in the caregiving situation (28). The most positive effects of these interventions have been exhibited during the COVID-19 pandemic (29, 30).

The findings of the present study showed that the support based on FCEM did not have a significant effect on the reduction of the care burden among family caregivers of patients with MS. Contrary to the present finding, the previous study conducted by Deyhoul et al. (9) revealed that implementing FCEM significantly reduces the care burden of patients with stroke (9). Moreover, the findings of the study by Shoghi et al. (11) indicated that such supportive intervention effectively reduces the care burden of caregivers of children with cancer (11). Other studies have been performed on caregivers of other chronic diseases (like Parkinson’s, hemodialysis, and epilepsy), whose results indicate the desired impact of this kind of support on the reduction of the negative consequences of care, such as care burden and psychological distress (28, 31, 32). The possible reasons for the contradiction in the findings can be considered since the FCEM intervention was implemented during the COVID-19 pandemic. Therefore, this factor may have shown the real impact of implementing a support intervention less than in times other than the COVID-19 pandemic (33). On the other hand, the post-test scores of care burden have been evaluated 1 month after the completion of the intervention, which may not show the long-term effects of this supportive intervention in this period. In addition, participants of this study were providing care to a chronic patient, so it’s possible to not observe the significant changes in the short term in the negative consequences of care (such as caregiving burden).

Families play a crucial role in meeting the care needs of patients due to disease or disability; however, their health and well-being may be endangered without sufficient support. Reviewing the scientific literature highlights the need to move toward family-centered care to enhance the well-being of disabled people and their family caregivers (34). According to the evidence, family-centered care may be vital to improving healthcare quality (35). Also, based on previous studies, providing support based on FCEM is effective in other caregiving aspects, such as improving the health literacy and self-efficacy of MS caregivers (36).

The current study had some limitations, such as the selection bias (37) and the lack of an approved standard scale to specify the care burden for caregivers of people with MS. The follow-up after the intervention was done only one month after the end of the intervention, so it is recommended to assess and follow up participants more than once and with a longer interval to examine the long-term effects of this intervention in future studies. Performing this study during the COVID-19 pandemic differentiates the findings from the previous studies. Since the intervention method was previously introduced for the Iranian community and in the current study, it was personalized and implemented according to the MS disease; it may have low generalizability to other conditions. Therefore, it is necessary to study its reproducibility in other settings (other cultures or different diseases, etc.). On the other hand, given the specific cultural and social norms in caring for patients in an Islamic country, the present results have limited generalization to Iranian caregivers. Due to the exclusion of caregivers with psychiatric diagnoses from this study, the clinical benefit of this study may have been reduced. Thus, it is recommended that this exclusion criterion be eliminated for a larger study in the future, which may lead the findings to align with clinical reality where there is psychiatric comorbidity (38).

The results of the present study indicate that FCEM does not have a favorable effect in reducing the care burden among caregivers of patients with MS. Therefore, it is recommended that FCEM be evaluated in different care contexts (such as culture, disease, etc.) with other possible long-term effects in future studies.

## Data availability statement

The raw data supporting the conclusions of this article will be made available by the authors, without undue reservation.

## Ethics statement

The studies involving human participants were reviewed and approved by Ethics Council in Biomedical Research of Mashhad University of Medical Sciences. The patients/participants provided their written informed consent to participate in this study.

## Author contributions

FM, SM, and HZ contributed to the conceptualization and designing of the study. FM, SM, and MM were involved in the data gathering and data cleaning. FM and HZ designed and performed data analysis and interpretation of the results. All authors contributed to the article and approved the submitted version.

## Funding

The present study resulted from a nursing thesis approved by the research council of Mashhad School of Nursing and Midwifery under referral code 991647.

## Conflict of interest

The authors declare that the research was conducted in the absence of any commercial or financial relationships that could be construed as a potential conflict of interest.

## Publisher’s note

All claims expressed in this article are solely those of the authors and do not necessarily represent those of their affiliated organizations, or those of the publisher, the editors and the reviewers. Any product that may be evaluated in this article, or claim that may be made by its manufacturer, is not guaranteed or endorsed by the publisher.
